# Midgut malrotation: a rare presentation of bowel obstruction in the adult

**DOI:** 10.1093/jscr/rjab309

**Published:** 2021-07-23

**Authors:** Ulanda Singh, Akil Baker

**Affiliations:** Department of Surgery, University Hospital of the West Indies, Mona, Jamaica; Department of General Surgery, University Hospital of the West Indies, Mona, Jamaica

## Abstract

Intestinal malrotation is a rare congenital abnormality. Usually, patients with malrotation of the intestine present in the neonatal period or the first year of life while some may remain asymptomatic and present later in childhood or adulthood. The diagnosis is usually delayed due to the sequence of events being that of non-specific gastrointestinal symptoms, which culminates in either adhesive bowel obstruction or volvulus. A 59-year-old male diagnosed with a large, incarcerated, right inguinoscrotal hernia underwent emergency laparotomy, which revealed midgut malrotation and small bowel obstruction due to Ladd bands. A modified Ladd’s procedure and right inguinal herniorrhaphy was performed.

## INTRODUCTION

Intestinal malrotation refers to the failure of the usual 270° counter clockwise rotation of the midgut around the superior mesenteric vessels during embryologic development [1]. Usually, patients with malrotation of the intestine present in the neonatal period or the first year of life while some may remain asymptomatic and present later in childhood or adulthood. Symptomatic cases occur in 1 in 6000 newborns [2] and 0.2% of the adult population [3]. The diagnosis is usually delayed due to the sequence of events being that of non-specific gastrointestinal symptoms, which culminates in either adhesive bowel obstruction or volvulus.

Here, an incidental finding of intestinal malrotation in an adult who had surgery for and incarcerated inguinoscrotal hernia is discussed.

## CASE

A 59-year-old man with no known chronic illnesses developed a right inguinal hernia 6 months prior to presentation. He presented to the emergency department with complaints of constipation and obstipation for 10 days, colicky abdominal pain and intermittent bilious vomiting. No history of fever was reported, he noted the hernia had increased in size significantly during this period and could no longer be manually reduced.

Examination findings revealed normal vital signs, chest examination was normal, the abdomen was grossly distended and a large right inguinoscrotal swelling which extended to mid-thigh was noted. No peristaltic waves could be seen within the inguinoscrotal swelling. There was mild right lower abdominal tenderness upon palpation, bowel sounds were increased and could be heard within the inguinoscrotal swelling.

The clinical diagnosis of complete bowel obstruction secondary to an incarcerated right inguinoscrotal hernia was made. This was supported by abdominal X-rays, which showed dilated small bowel with multiple air fluid levels.

**Figure 1:**
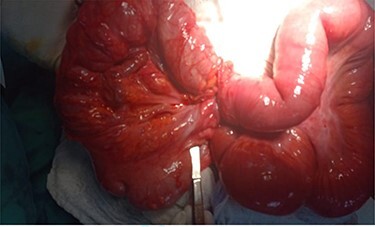
Contents of the hernia sac: cecum and ascending colon (right), herniated ileum through mesenteric defect (left).

**Figure 2:**
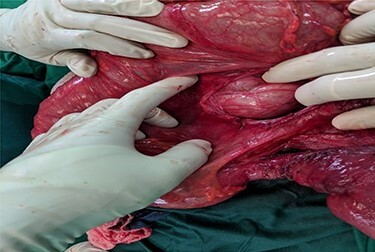
Mesenteric defect seen after ileum was reduced.

**Figure 3:**
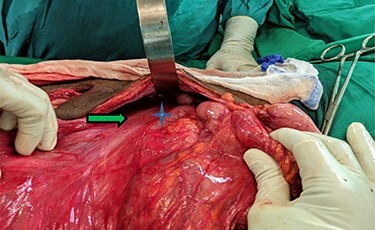
Narrow based, unfixed mesentery of right colon (arrow) with a right sided DJ flexure ^*^.

Hernia reduction was attempted first through a right inguinal incision, which was unsuccessful. A lower midline laparotomy was then performed which revealed viable ileum, cecum, ascending colon within the hernia sac, and multiple adhesive bands (Ladd’s bands). A mesenteric defect through which the ileum herniated (Figs 1 and 2) and was also noted to be the point of obstruction. The duodeno-jejunal (DJ) flexure was noted to be on the right of the superior mesenteric artery (SMA) (Fig. 3). The left colon was normally oriented and fixed onto the posterior abdominal wall.

**Figure 4:**
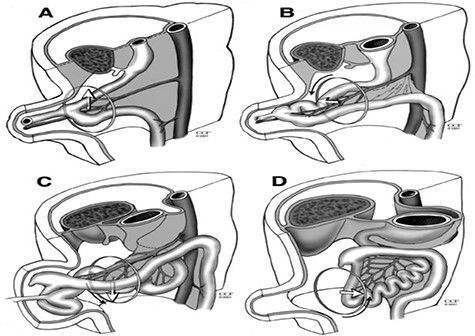
Diagram of normal intestinal rotation. (**A**) Primitive gut, (**B**) stage 1, (**C**) stage 2 and (**D**) stage 3.

A diagnosis of intestinal malrotation with incomplete fixation was made. A modified Ladds’ procedure was done. This included lysis of Ladds’ bands, fixation of the hepatic flexure and repair of the mesenteric defect. An appendectomy was not done since it was now within the right lower quadrant after fixation of the hepatic flexure. The post-operative period was un-eventful and the patient was discharged from hospital on post-operative day three.

## DISCUSSION

Embryologic midgut rotation occurs when the mesentery of the bowel folds around the SMA axis in a counter clockwise manner and there is fixation of the mesentery to the peritoneal cavity [4]. This process occurs in three stages, beginning in the fifth week of life and continues until full maturity [1]. The embryologic midgut starts off as a vertical loop parallel to the SMA axis, the cranial end being the DJ loop and caudal end the ceco- colic (C-C) loop. It then rotates as follows: (Fig. 4)

- Stage 1: occurs between 5 and 10 weeks; there is herniation of the previously vertical intestinal loops into the umbilical defect and an initial rotation of 90 degrees counter clockwise around the SMA axis. This places the DJ loop to the right of the SMA and the C-C loop to the left.
- Stage 2: occurs between 11 and 12 weeks, an additional 180° rotation occurs placing the DJ loop to the left of the SMA, C-C loop is to the right and superior to the SMA.
- Stage 3: occurs from 12 weeks onward; there is fixation of the mesentery such that the C-C loop is now on the right of the SMA.

Deviation from this normal pattern of herniation, counter clockwise pattern of rotation and fixation to the posterior abdominal wall results in malrotation or incomplete fixation. This rare phenomenon usually presents within the first month of life [6, 7], the incidence of which ranges from 1 in 200 to 500 live births. [8, 1] An even rarer presentation is malrotation in the adult, the incidence of which is 0.2% [9]. In Jamaica, there is no documented case of intestinal malrotation in adults. However, there have been cases noted in children [10].

Adults with intestinal malrotation usually have a prolonged history of non-specific, recurrent abdominal pain which results in frequent visits to the doctor and no diagnosis found. Dietz *et al*. described two sets of patients; those with chronic obstructive symptoms and those with acute obstructive symptoms [5]. It is not until acute obstruction occurs that malrotation is diagnosed either via imaging or at laparotomy. Acute presentations are caused by midgut volvulus or adhesive obstruction via Ladds’ bands [ 2]. Patients have also been known to present with internal hernias. Our patient presented with an acute bowel obstruction via an obstructing internal hernia resulting in abdominal distension, enlargement and irreducibility of the inguinal hernia. Review of literature from the last 16 years revealed no such documented case.

The gold standard for diagnosis of malrotation is upper GI series or CT with oral and IV contrast. In adults, CT is preferred. [2, 11] There are a few features which can be seen that cinches the diagnosis such as the ‘whirl pool’ sign seen in intestinal volvulus; the inverse relationship (SMV rotation sign) between the superior mesenteric vein (SMV) and artery (SMA), where the SMV lies anterior and to the right of the SMA [12] There is also right sided small bowel, a right sided DJ flexure and cecum to the left [8]. Garcelan *et al*. [9] also described the ‘Barber pole’ sign which is whirling of the SMA and its branches. We did no CT abdomen for our patient since he clinically had clear evidence of intestinal obstruction which required surgical correction.

Management of intestinal malrotation has been described by Dr William Ladd in 1936, his surgical procedure; the Ladd procedure, which remains the corner stone of treatment [13]. This involves lysis of Ladds’ bands, placement of the small bowel on the right, cecum in the left upper or lower quadrant and appendectomy. Our patient had a modified Ladd’s procedure since he had the usual counter clockwise rotation with incomplete fixation of the right colon. No appendectomy was done since after fixation of the hepatic flexure the appendix was now in anatomical position and the dilemma of a wandering appendix would not be present should he develop acute appendicitis in the future. Another reason for appendectomy is lysis of Ladds’ bands can potentially devascularize the appendix. Our patient had few bands so the decision was made to forego the appendectomy.

## CONCLUSION

The diagnosis of intestinal malrotation is not usually considered as a cause of bowel obstruction in adults because of its rarity. More often these patients have non-specific abdominal symptoms for which no diagnosis can be found until they present with a complication such as volvulus or bowel ischemia. Hence, a high index of suspicion is needed.

## CONFLICT OF INTEREST STATEMENT

None declared.

## FUNDING

None.
